# Muscle acetylcholine receptor conversion into chloride conductance at positive potentials by a single mutation

**DOI:** 10.1073/pnas.1908284116

**Published:** 2019-09-30

**Authors:** Hakan Cetin, Max Epstein, Wei W. Liu, Susan Maxwell, Pedro M. Rodriguez Cruz, Judith Cossins, Angela Vincent, Richard Webster, Philip C. Biggin, David Beeson

**Affiliations:** ^a^Nuffield Department of Clinical Neurosciences, University of Oxford, Oxford OX3 9DS, United Kingdom;; ^b^Department of Neurology, Medical University of Vienna, 1090 Vienna, Austria;; ^c^Structural Bioinformatics and Computational Biochemistry, Department of Biochemistry, University of Oxford, Oxford OX1 3QU, United Kingdom

**Keywords:** myasthenia, acetylcholine receptor, charge selectivity

## Abstract

Charge selectivity forms the basis of cellular excitation or inhibition by Cys-loop ligand-gated ion channels (LGICs), and is essential for physiological receptor function. There are no reports of naturally occurring mutations in LGICs associated with the conversion of charge selectivity. Here, we report on a CHRNA1 mutation (α1Leu251Arg) in a patient with congenital myasthenic syndrome associated with transformation of the muscle acetylcholine receptor (AChR) into an inhibitory channel. Performing patch-clamp experiments, the AChR was found to be converted into chloride conductance at positive potentials, whereas whole-cell currents at negative potentials, although markedly reduced, were still carried by sodium. Umbrella sampling molecular dynamics simulations revealed constriction of the channel pore radius to 2.4 Å as a result of the mutation, which required partial desolvation of the ions in order to permeate the pore. Ion desolvation was associated with an energetic penalty that was compensated for by the favorable electrostatic interaction of the positively charged arginines with chloride. These findings reveal a mechanism for the transformation of the muscle AChR into an inhibitory channel in a clinical context.

## Significance

We report on a single mutation in the α1-subunit M2 helix (p.α1Leu251Arg) of the muscle acetylcholine receptor (AChR) found in a patient with congenital myasthenic syndrome (CMS) that is shown to convert the AChR into chloride conductance at positive potentials. Constriction of the channel pore with partial desolvation and stabilization of the permeating chloride ions by the arginine residues is revealed as the underlying mechanism. This article is of general interest because it describes a mechanism for the transformation of the muscle AChR into an inhibitory channel, and presents a report of charge selectivity conversion in association with a naturally occurring single mutation. Our findings might also give explanation to a pathomechanism in CMS.

The nicotinic acetylcholine receptor (AChR) in muscle is a member of the Cys-loop ligand-gated ion channel (LGIC) superfamily. It mediates electrical signal transmission from nerve to muscle via the opening/closing of a transmembrane cation conductive pore. The AChR is found at high density on the postsynaptic membrane of the neuromuscular junction, where it generates the endplate potential (EPP) in response to acetylcholine (ACh) released from the nerve terminal into the synaptic cleft. The EPP usually exceeds the threshold potential required for the activation of voltage-gated sodium channels that initiate an action potential in the muscle fiber and trigger muscle contraction. Various congenital myasthenic syndromes (CMSs) have been attributed to a malfunction of AChRs. The mutations reported to date affect different receptor properties such as ligand binding, conductance, gating, or desensitization (1, 2), but none have been shown to change ion selectivity.

The AChR channel pore selects for cations according to size (3) and is formed by the second transmembrane (M2) helix of each of the 5 receptor subunits (4). Cation selectivity is determined by the charge distribution along the ion permeation pathway. The intracellular and extracellular vestibules of the receptor adjacent to the transmembrane pore are electronegative and provide an environment to stabilize cations and increase their local concentration (5, 6). The upper portion of the pore is lined with hydrophobic residues restricting water occupancy and, as a result, prevents the passage of ions in the closed state (i.e., the hydrophobic gating mechanism) (7, 8). Upon receptor activation, the pore widens due to a displacement of the M2 helices, which relieves the hydrophobic gate and allows the hydration of the pore and the conduction of ions (9, 10). Within the pore, ions are further stabilized by interactions with ionized side chains in the first turn of the M2 α-helices (11). At the bottom of the transmembrane pore, negatively charged residues also facilitate cation entry (8, 12).

Charge selectivity is essential for physiological receptor function and forms the basis for excitatory and inhibitory LGICs. Mutations associated with reversed charge selectivity would give rise to transmembrane potentials that are physiologically detrimental. The first evidence for a transformation of a cation-selective into an anion-selective LGIC derived from experiments on chimeric α7-neuronal AChRs with substituted homologous residues from the glycine receptor (13). A minimum number of 2 substitutions (E237A and V251T) and the insertion of a proline residue between positions G236 and E237 were necessary for a successful transformation. The introduction of the same 3 mutations into 5-hydroxytryptamine_3_ receptors again resulted in a cation-to-anion transformation of selectivity (14). Similar effects were observed in the glycine receptor after the introduction of the 3 inverse mutations from the α7-neuronal AChR, by which selectivity was switched from anionic into cationic (15). Together, these experimental constructs reveal that the substitution of a number of key residues in the M2 helix of LGICs is sufficient to mediate the conversion of charge selectivity, which, however, has not been reported in association with naturally occurring mutations and disease.

Here, we present functional and structural data on a CHRNA1 mutation (p.Leu251Arg) identified in a patient with CMS. The mutation is located within the highly conserved M2 transmembrane helix, which lines the channel pore. This study provides evidence for a voltage-dependent conversion of ion selectivity from cationic to anionic and, consequently, the transformation of the muscle AChR from an excitatory into an inhibitory channel caused by a single amino acid substitution.

## Results

### Clinical Features of the Patient with Congenital Myasthenic Syndrome.

The patient, now 14 y old, is female. Onset of symptoms was in infancy with feeding difficulties, dribbling, and reduced facial expression, delay in motor milestones, clear bilateral fatigable ptosis, limited ocular movement, and marked restriction in upgaze. Muscle MRI was normal. A muscle biopsy was nondiagnostic, with no mitochondrial abnormality evident and respiratory chain enzyme analysis normal, but it did show some type II fiber atrophy. Electromyography showed decrement and jitter, suggesting a disorder of neuromuscular transmission. On recent examination the fatigable ptosis and restriction of eye movements were present, along with neck flexor and mild facial weakness. There was no obvious weakness in upper and lower limbs, though muscle weakness was reported after sustained walking or toward the end of the day.

The clinical features and electromyography results suggested a diagnosis of myasthenia. Assays for anti-AChR antibodies, including for clustered AChRs (16) and anti-MuSK antibodies were negative, and so screening of genes for mutations that could cause CMS was undertaken. Mutations were excluded in most CMS genes (including CHRNB1, CHRNE, CHRND, DOK7, RAPSN, DPAGT1, and GFPT1) but a single heterozygous de novo variant predicted to be pathogenic was identified in CHRNA1 (c.812T > G, p.Leu271Arg) (transcript numbering according to ENST00000348749.9, which does not harbor the P3A exon, but contains the 20 amino acid signal sequence; thus the mutation is at position Leu251Arg excluding the signal sequence). For ease of reference with the many previous reports on defining the function of residues within the AChR M2 helix, we will use the amino acid numbering according to the mature α1-subunit polypeptide and thus, from here, term this variant α1L251R. The CHRNA1 variant c.812T > G, p.L271R is not present on the exome variant server (https://evs.gs.washington.edu/EVS/) or in Ensembl genome browser 94 [(EMBL-EBI, Cambridge, UK (URL: https://www.ensembl.org)]. Screening of family DNA showed that this variant was not present in either parent, suggesting it arose de novo.

The p.α1L251 residue lies within the M2 helix of the AChR α1-subunit at the 9′ position corresponding to the consensus numbering system of Miller (17), in which position 1′ refers to the cytoplasmic end of the M2 helix. The consensus numbering system by Miller is used for comparison between different LGICs. The p.α1L251 residue is highly conserved across species and paralogues (18).

### Surface Expression of AChR_α1L251R_ Is Reduced.

**Fig. 1.**
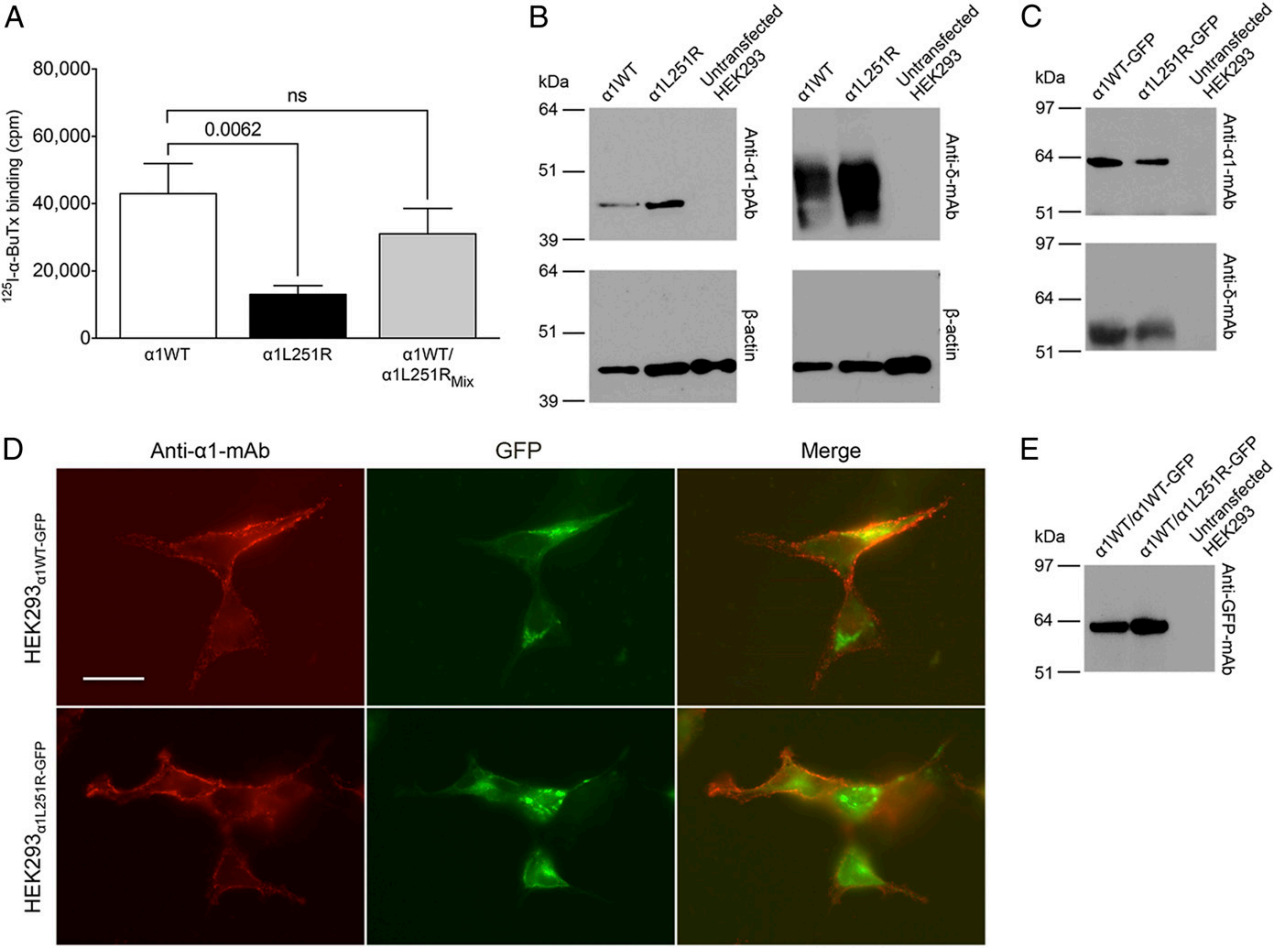
The influence of the α1L251R mutation on AChR surface expression. (*A*) The bars show final results of ^125^I-α-BuTx surface binding with the background level of radioactivity already subtracted from the cell-surface ^125^I-α-BuTx activity in transfected cells (*n* = 8 experiments). Data are mean ± SEM. (*B*) Western blots of whole-cell extracts from HEK293_α1WT_, HEK293_α1L251R_, and untransfected HEK293 cells probed with either a rabbit anti-human α1-polyclonal antibody (pAb) (*Left*) or a mouse anti-human δ- monoclonal antibody (mAb) (*Right*). β-Actin probed with a monoclonal antibody was used as a loading control. (*C*) Western blots of immunoprecipitated surface AChRs using a mouse anti-human α1-monoclonal antibody (*Top*) and a mouse anti-human δ-monoclonal antibody (*Bottom*), respectively. HEK293 cells were transfected with a 2:1:1:1 ratio of α1WT-GFP-, β1-, δ-, ε-subunits (*Left* lane) or with a 2:1:1:1 ratio of α1L251R-GFP-, β1-, δ-, ε-subunits (*Middle* lane). (*D*) Immunofluorescence images of HEK293 cells transfected with a 2:1:1:1 ratio of α1WT-GFP-, β1-, δ-, ε-subunits (*Top*) or with a 2:1:1:1 ratio of α1L251R-GFP-, β1-, δ-, ε-subunits (*Bottom*). (Scale bar, 20 μm.) (*E*) Western blots of immunoprecipitated surface AChRs using a mouse anti-GFP monoclonal antibody. HEK293 cells were transfected with a 1:1:1:1:1 ratio of α1WT-, α1WT-GFP, β1-, δ-, ε-subunits and a 1:1:1:1:1 ratio of α1WT-, α1L251R-GFP, β1-, δ-, ε-subunits. ns, not significant.

The impact of the α1L251R mutation on receptor surface expression was tested by transfection of cDNAs encoding the corresponding AChR subunits into HEK293 cells and counting AChR binding of radioactive ^125^I-α-bungarotoxin (^125^I-α-BuTx). Surface expression of AChR_α1L251R_ was reduced to ∼30.3% of AChR_α1WT_ (t_(14)_ = 3.2, *P* = 0.0062) (Fig. 1*A*). Detection of ^125^I-α-BuTx binding on the surface of the cells indicates that AChR_α1L251R_ is expressed on the cell surface, but for confirmation of this we used a monoclonal antibody to the extracellular main immunogenic region located on the AChR α1-subunit both for extracellular fluorescent labeling of AChRs (*SI Appendix*, Fig. S1) and in pull-down assays. The AChR_α1L251R_ is efficiently expressed in the cell lysate (Fig. 1*B* and *SI Appendix*, Fig. S2 *A* and *B*) but less well assembled and transported to the cell surface (Fig. 1 *C* and *D* and *SI Appendix*, Fig. S2*C*), explaining the reduction of ^125^I-α-BuTx binding to ∼30.3% of AChR_α1WT_. However, the AChR_α1L251R_ pentamer subunit composition appeared not to be altered as shown for the α1L251R-/δ-subunit ratio, which was similar to the α1WT-/δ-subunit ratio (Fig. 1*C* and *SI Appendix*, Fig. S2*D*). The ^125^I-α-BuTx binding to the surface of HEK293_α1WT/α1L251R_Mix_ cells transfected with a mixture of AChR_α1WT_ and AChR_α1L251R_ was not significantly different compared to HEK293_α1WT_ cells transfected with AChR_α1WT_ alone (Fig. 1*A*), with significant surface expression levels of the α1L251R subunit in the presence of the α1WT subunit (Fig. 1*E*).

### Whole-Cell Current Amplitude, Desensitization, and Deactivation Are Altered in Mutant AChRs.

**Fig. 2.**
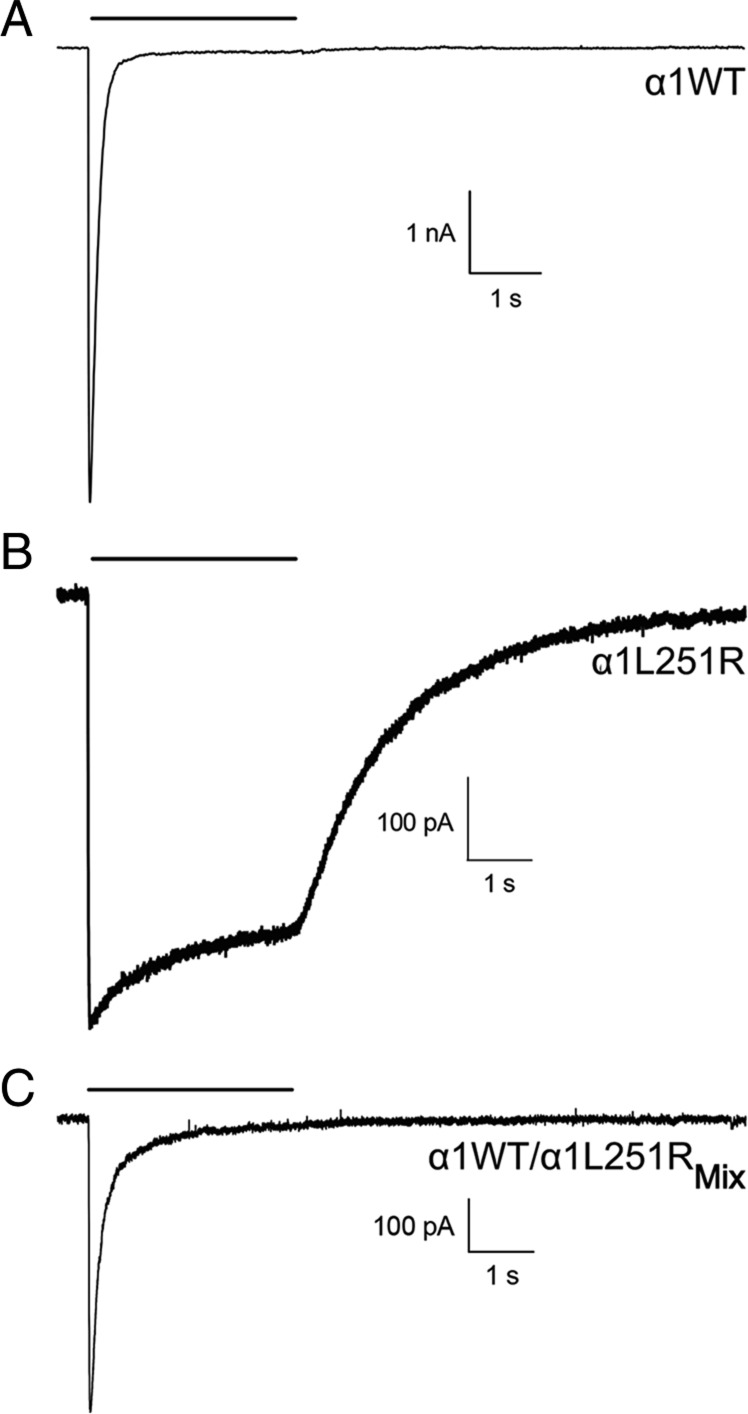
Current recordings of wild-type and mutant AChRs expressed in HEK293 cells. Compared to HEK293_α1WT_ (*A*), current amplitudes were lower in HEK293_α1L251R_ (*B*) and HEK293_α1WT/α1L251R_Mix_ (*C*). Desensitization and deactivation were markedly slower in HEK293_α1L251R_ compared to HEK293_α1WT_ and HEK293_α1WT/α1L251R_Mix_.

**Fig. 3.**
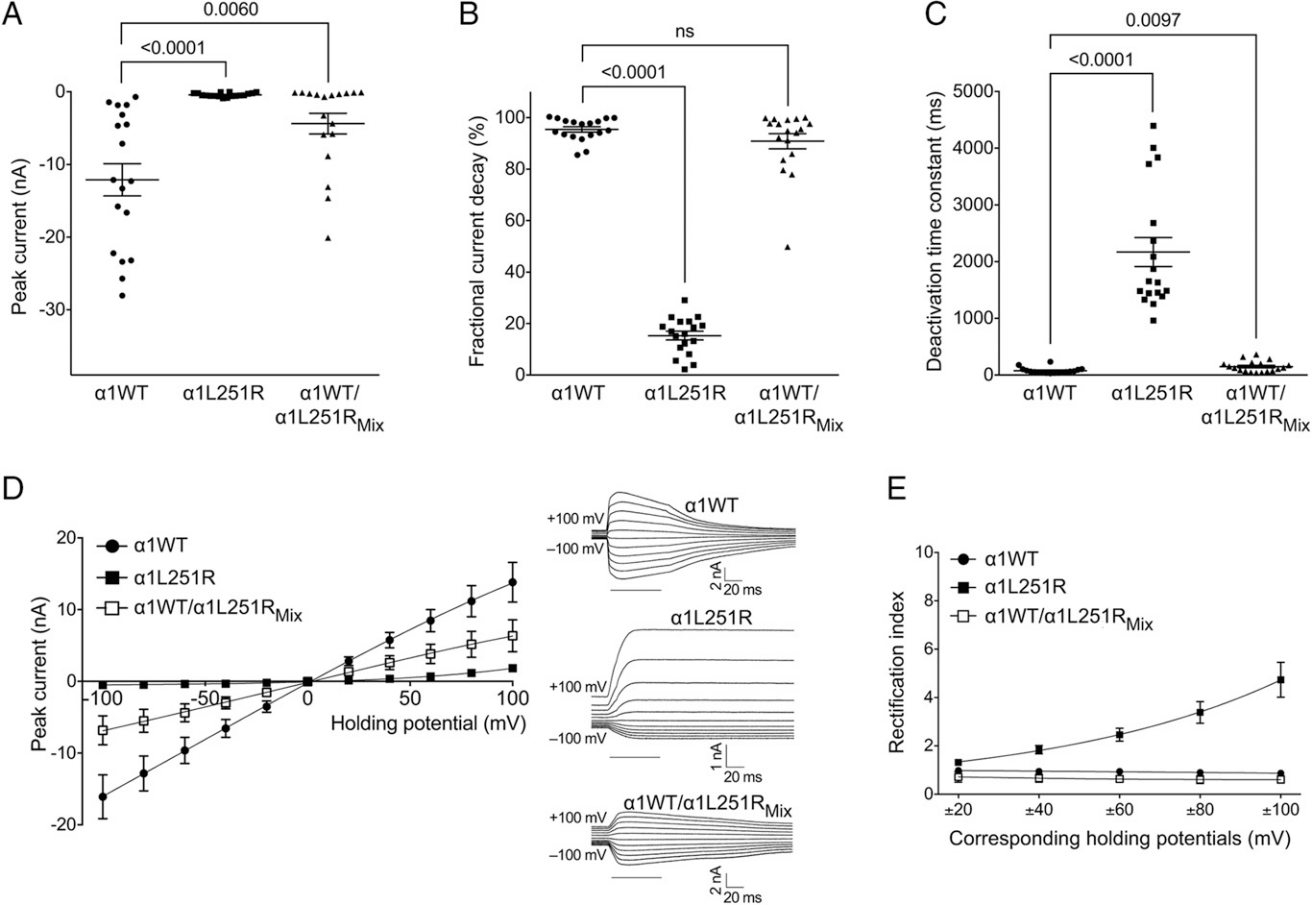
Functional characterization of wild-type and mutant AChRs expressed in HEK293 cells. The α1L251R mutation was associated with reduced peak currents (*A*), slower desensitization (*B*) and deactivation (*C*) and with a change of the linear I–V relationship into outward rectification in HEK293_α1L251R_ (*D*) and a RI > 1 at several holding potentials (*E*). HEK293_α1WT_: *n* = 16, HEK293_α1L251R_: *n* = 17, HEK293_α1WT/α1L251R_Mix_: *n* = 15. Data are mean ± SEM. ns, not significant.

The functional consequences of the α1L251R mutation were first assessed by the analysis of burst duration of mutant channels transiently expressed in HEK293 cells. Performing recordings at low (100 nM) and high (30 μM) ACh concentrations and at various holding potentials ranging between −100 mV and +80 mV, no channel activity could be detected (5 transfections and 50 patches tested). Although surface expression levels of AChR_α1L251R_ were reduced, the lack of any channel activity was unusual. To further evaluate the functional consequences of the α1L251R mutation, we then performed whole-cell patch-clamp experiments. In AChR_α1WT_, current traces displayed a fast rise and a peak followed by an exponential current decay with almost complete desensitization after a 3-s pulse of 1 mM ACh (Fig. 2*A*). In AChR_α1L251R_, current shape was changed in many aspects (Fig. 2*B*). Current amplitude was significantly reduced (by 96.7%) in AChR_α1L251R_ (*n* = 18) compared to AChR_α1WT_ (*n* = 18) (mean current amplitude of −12.1 ± 2.2 nA in AChR_α1WT_ and −0.4 ± 0.1 nA in AChR_α1L251R_, t_(34)_ = 5.3, *P* < 0.0001) (Fig. 3*A*), and desensitization was markedly reduced (mean fractional current decay of 95.4 ± 1.0% in AChR_α1WT_ and 15.3 ± 1.7% in AChR_α1L251R_, t_(34)_ = 40.3, *P* < 0.0001) (Fig. 3*B*). Receptor deactivation, defined as current decay during ACh washout at the end of receptor stimulation when remaining open receptors return to the closed state, was slower in AChR_α1L251R_ compared to AChR_α1WT_ (mean deactivation time constant of 76 ± 13 ms in AChR_α1WT_ and 2,169 ± 256 ms in AChR_α1L251R_, t_(34)_ = 8.0, *P* < 0.0001) (Fig. 3*C*).

In HEK293_α1WT/α1L251R_Mix_ cells expressing AChRs with a mixed range of subunit stoichiometries, current amplitude was significantly reduced compared to HEK293_α1WT_ (−12.1 ± 2.2 nA in HEK293_α1WT_ and −4.4 ± 1.4 nA in HEK293_α1WT/α1L251R_Mix_, t_(34)_ = 2.9, *P* = 0.0060) (Fig. 3*A*). Current decay was similar comparing HEK293_α1WT/α1L251R_Mix_ with HEK293_α1WT_ (mean fractional current decay of 95.4 ± 1.0% in HEK293_α1WT_ and 90.8 ± 2.9% in HEK293_α1WT/α1L251R_Mix_, t_(34)_ = 1.5, *P* = 0.1512) (Fig. 3*B*) but deactivation was significantly slower (mean deactivation time constant of 76 ± 13 ms HEK293_α1WT_ and 148 ± 23 ms in HEK293_α1WT/α1L251R_Mix_, t_(34)_ = 2.7, *P* = 0.0097) (Fig. 3*C*).

The comparison of the current–voltage (I–V) relationship between HEK293_α1WT_, HEK293_α1L251R_, and HEK293_α1WT/α1L251R_Mix_ revealed further functional aspects of the mutation. Whereas the I–V relationship was linear in HEK293_α1WT_ and HEK293_α1WT/α1L251R_Mix_ with a rectification index (RI) close to 1, there was a strong outward rectification observed at positive potentials in HEK293_α1L251R_ with an increasing RI of up to 4.7 ± 0.7 at ±100 mV (Fig. 3 *D* and *E*). There was no shift of the reversal potential.

### The αL251R Mutation Is Associated with a Conversion from Cationic to Anionic Selectivity.

**Fig. 4.**
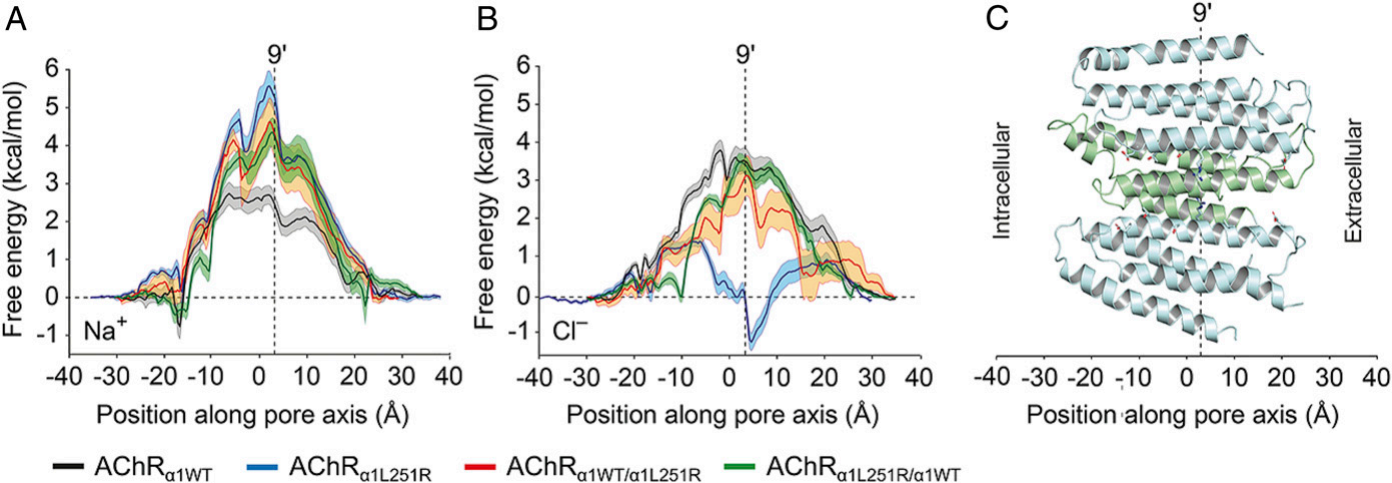
Analysis of Na^+^ and Cl^–^ passage through the transmembrane pore in wild-type and mutant AChRs by PMF calculations. PMF profiles for Na^+^ (*A*) and Cl^−^ (*B*) ions in AChR_α1WT_ (black), AChR_α1L251R_ (blue), AChR_α1WT/α1L251R_ (red), and AChR_α1L251R/α1WT_ (green). (*C*) View on side of AChR_α1L251R_ model within the same coordinate space as the PMF profiles in *A* and *B*. Residues at key positions are indicated in stick representation. The β1- and δ-subunits have been removed for clarity. At AChR_α1L251R_, the reduction of the chloride barrier height to 1.5 kcal/mol around −8 Å as well as the introduction of an energetic well of −1.15 kcal/mol around 5 Å (together with the increase in barrier height to 5.57 kcal/mol for sodium) is indicative of strong chloride selective permeation. Error bars are 1 SD of the mean.

Electrophysiological experiments were performed at different AChR surface expression levels in HEK293_α1WT_ and HEK293_α1L251R_. However, the whole-cell current reduction in AChR_α1L251R_ to 3.3% of AChR_α1WT_ in HEK293 cells was not explained by the surface expression, which was reduced to 30.3% in AChR_α1L251R_ compared to AChR_α1WT_. A functional defect of mutant AChRs, therefore, was a theory also supported by the altered whole-cell current kinetics in HEK293_α1L251R_ and the significantly reduced whole-cell current amplitudes to 36.4% in HEK293_α1WT/α1L251R_Mix_, in which AChR surface expression was not statistically different compared to HEK293_α1WT_ (Fig. 1*A*). We performed umbrella sampling molecular dynamics (USMD) simulations of wild-type and mutant AChRs to investigate the influence of the mutation on sodium and chloride passage through the transmembrane pore. First, potentials of mean force (PMFs) for AChR_α1WT_ were computed for both sodium and chloride ions (Fig. 4 *A*–*C*). The computed PMFs for AChR_α1WT_ indeed demonstrated selectivity for sodium, as expected. All barrier heights for sodium at mutant channels were higher than for chloride at AChR_α1WT_, indicative of reduced sodium permeability, and both AChR_α1WT/α1L251R_ and AChR_α1L251R/α1WT_ possessed similar (within error) PMF profiles.

Chloride barrier heights at AChR_α1WT/α1L251R_/AChR_α1L251R/α1WT_ were greater than that for sodium at AChR_α1WT_ but lower than that for chloride at AChR_α1WT_. Taken together with the experimental results above, it is likely that these values represent nonconductive states. It is worth noting that there was some discernible difference between the overall shapes of the PMF energy landscapes at AChR_α1WT/α1L251R_/AChR_α1L251R/α1WT_, likely arising from the pseudosymmetrical arrangement of subunit stoichiometries. However, this is unlikely to result in meaningful physiological differences between these channels.

**Fig. 5.**
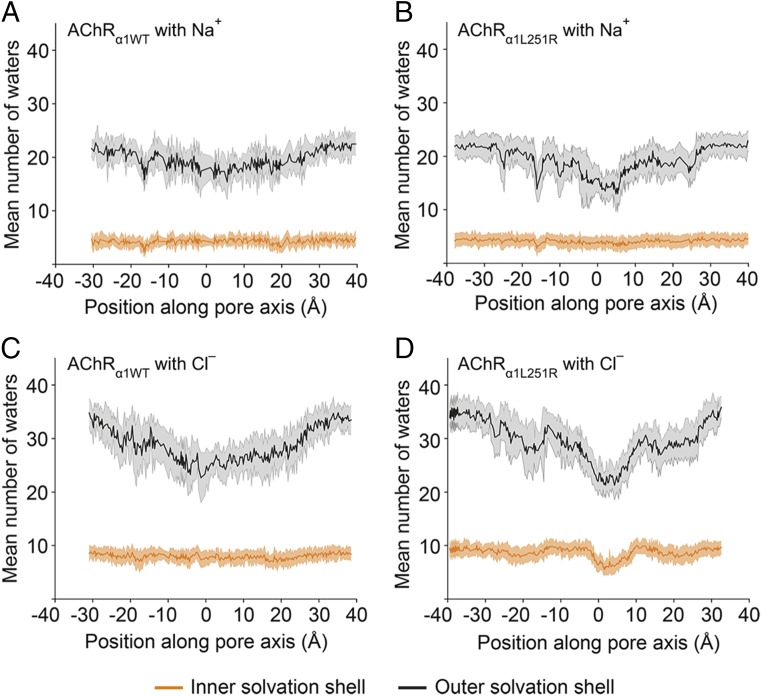
Solvation shells of Na^+^ and Cl^−^ ions as a function of their position along the reaction coordinate. Sodium is never desolvated from its inner solvation shell and only minor perturbations are observed in the outer solvation shell in both AChR_α1WT_ and AChR_α1L251R_ (*A* and *B*). Thus, the lack of Na^+^ permeation observed reflects repulsion of the solvated Na^+^. By contrast, Cl^−^ is observed to be desolvated around the L251R residue (around 3 Å in the reaction coordinate) in AChR_α1L251R_ (*D*) but not in AChR_α1WT_ (*C*). Error bars represent 1 SD.

**Fig. 6.**
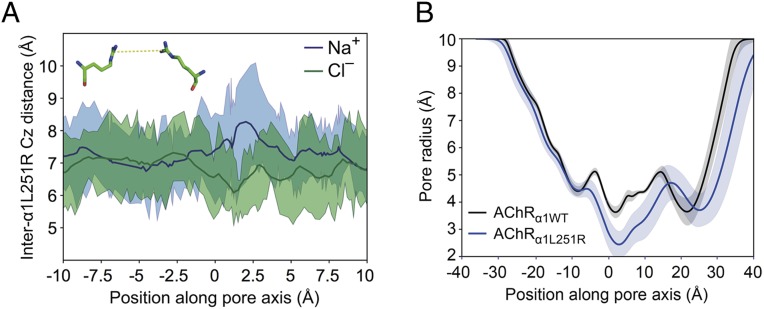
Comparison of AChR_α1L251R_ guanidinium carbon internuclear distances with respect to either Na^+^ or Cl^–^ permeation, and pore radii comparison between AChR_α1WT_ and AChR_α1L251R_. (*A*) Internuclear distances between central guanidinium carbons of both α1L251R residues show that Cl^−^ pulls the residues closer together at ∼1.25 Å along the reaction coordinate, with Na^+^ exhibiting an opposing effect. Carbon is denoted by the forcefield atom type notation “*Cz*.” (*B*) The narrow 2.4-Å pore radius of α1L251R near 2.5 Å in the reaction coordinate provides further evidence of tight coordination of Cl^−^ with α1L251R residues. Indeed, Fig. 5*D* demonstrates the partial dewetting that would need to occur in order to accommodate a permeating ion through a channel of this diameter. Error bars represent 1 SD.

The most pronounced effect occurred for chloride in AChR_α1L251R_, revealing a reduction in barrier height to 1.5 kcal/mol (around −8 Å) as well as the introduction of an energetic well around *z* = 5 Å of −1.15 kcal/mol. This, together with the increase in barrier height to 5.57 kcal/mol for sodium is indicative of strongly chloride selective permeation. Analyses of the first and second solvation shells (Fig. 5) of chloride at AChR_α1L251R_ showed that desolvation of these shells occurs around position *z* = 3 Å in the reaction coordinate, concordant with the reduction in channel radius at this location. The energetic penalty incurred through this desolvation is accounted for by the favorable electrostatic interactions of both arginine residues with chloride, in which they act as surrogate water molecules chelating the probe ion (Fig. 6*A*). An energetic well in the permeation pathway would be expected to slow down passage of ions and may contribute to a lower conductance than would be predicted on the basis of diameter alone.

Both chloride and sodium possess ion-O internuclear distances within their first solvation shell (19) (Fig. 6*A*) that would be incompatible for conduction through a pore of the size seen in AChR_α1L251R_ (radius of ∼2 Å) (Fig. 6*B*). In trajectories from the PMF profiles, sodium was never desolvated from its first solvation shell (Fig. 5 *A* and *B*) and only minor perturbations were observed in the second solvation shell in both AChR_α1WT_ and AChR_α1L251R_. Thus, the lack of permeation observed reflects repulsion of the solvated sodium ion. By contrast, chloride was observed to be desolvated (with a more substantial deformation of the second solvation shell) around the L251R residues (9′ position) in AChR_α1L251R_ but not in AChR_α1WT_ (Fig. 5 *C* and *D*).

### The Conversion from Cationic to Anionic Selectivity Is Voltage Dependent.

**Fig. 7.**
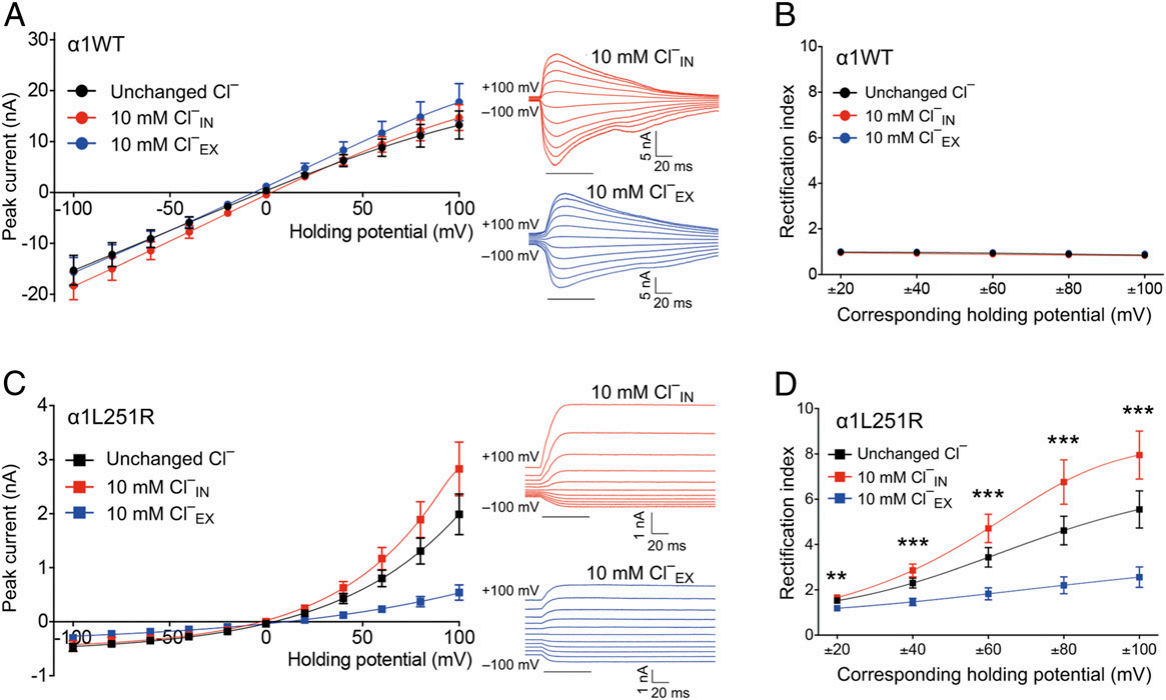
The I–V relationship in AChR_α1WT_ and AChR_α1L251R_ in dependence of the intracellular (_IN_) and extracellular (_EX_) Cl^–^ concentration. In AChR_α1WT_, the I–V relationship was independent of the intra- or extracellular Cl^−^ concentration (*A*), with a RI around 1 in all groups with different Cl^−^ gradients (*B*), suggesting insignificant Cl^−^ conductance (unchanged Cl^−^: black, *n* = 17; 10 mM Cl^−^_IN_: red, *n* = 11; 10 mM Cl^−^_EX_: blue, *n* = 10). In AChR_α1L251R_, the I–V relationship was independent of the intra- or extracellular Cl^−^ concentration at negative holding potentials, but was strongly chloride dependent at positive holding potentials (*C*), when current amplitudes increased at a reduced intracellular Cl^−^ concentration and decreased at a reduced extracellular Cl^−^ concentration, indicating a significant Cl^−^ conductance in AChR_α1L251R_ at positive holding potentials only (unchanged Cl^−^: black, *n* = 16; 10 mM Cl^−^_IN_: red, *n* = 11; 10 mM Cl^−^_EX_: blue, n =12). The RI displayed a sigmoidal relationship in all groups with differing Cl^−^ gradients (*D*). Data are mean ± SEM. **P < 0.01; ***P < 0.001 one-way ANOVA with Tukey’s post hoc correction to test for a difference between 10 mM Cl^−^_IN_ and 10 mM Cl^−^_EX_.

USMD simulations were complemented by whole-cell patch-clamp experiments with altered Cl^−^ and NaCl concentrations, respectively, in order to elucidate current rectification and the conversion of ion selectivity associated with the α1L251R mutation. The I–V relationship was analyzed after substitution of intra- or extracellular Cl^−^ by SO_4_^2−^ to a residual Cl^−^ concentration of 10 mM. In AChR_α1WT_, Cl^−^ substitution had no appreciable effect on current rectification and reversal potential (Fig. 7 *A* and *B*). In AChR_α1L251R_, by contrast, the reduction of intracellular Cl^−^ concentration was associated with increased current amplitudes at positive potentials with the RI increasing to 8.0 ± 1.1 at ±100 mV (Fig. 7 *C* and *D*). Correspondingly, the reduction of extracellular Cl^−^ concentration reduced current amplitudes at positive potentials with a RI of 2.6 ± 0.4 at ±100 mV. Reversal potential was not shifted and AChR kinetics not affected by either condition in AChR_α1L251R_, suggesting no significant Cl^−^ permeability at negative potentials up to 0 mV.

**Fig. 8.**
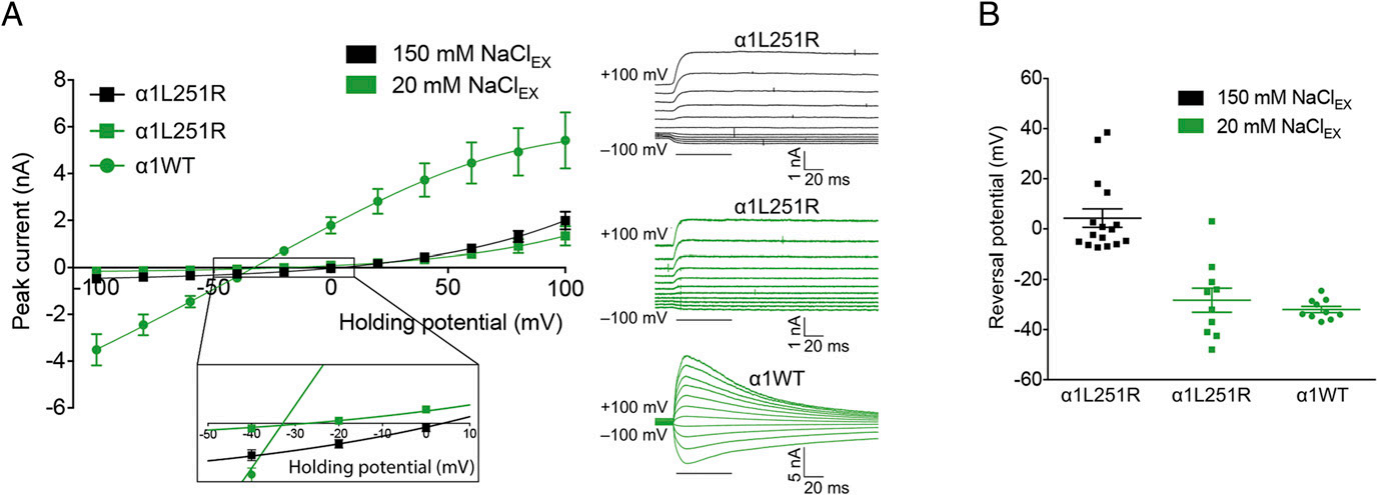
The I–V relationship in AChR_α1WT_ and AChR_α1L251R_ in dependence of the extracellular NaCl concentration. The substitution of extracellular NaCl by glucose to a residual concentration of 20 mM resulted in a shift of the reversal potential toward negative voltages following the equilibrium potential of potassium in AChR_α1WT_ (*A*). In AChR_α1L251R_, the shift of the reversal potential upon substitution of extracellular NaCl by glucose followed the equilibrium potential of potassium toward negative voltages (*A*), with no significant difference of the reversal potential between AChR_α1L251R_ and AChR_α1WT_ (*B*). Data are mean ± SEM.

To explore a voltage-dependent conversion of ion selectivity in AChR_α1L251R_ further, we performed experiments in which extracellular NaCl was substituted by glucose to a residual concentration of 20 mM. This resulted in a shift of the reversal potential toward negative voltages in AChR_α1L251R_ following the equilibrium potential of potassium (reversal potential of −28.3 ± 4.8 mV at 20 mM extracellular NaCl [*n* = 10] compared to 4.3 ± 3.7 mV at 150 mM extracellular NaCl [*n* = 16], t_(24)_ = 5.4, *P* < 0.0001), and was very similar to the shift observed in AChR_α1WT_ (reversal potential of −28.3 ± 4.8 mV in AChR_α1L251R_ at 20 mM NaCl [*n* = 10] compared to −32.0 ± 1.3 mV in AChR_α1WT_ at 20 mM NaCl [*n* = 10], t_(18)_ = 0.7, *P* = 0.4649) (Fig. 8 *A* and *B*). This is indicative of negligible chloride permeability at negative membrane potentials in AChR_α1L251R_. Together, these findings provide evidence for a significant contribution of chloride ions to AChR_α1L251R_ currents at positive potentials only.

## Discussion

The present study describes the effects of a mutation, α1L251R, found in a patient with CMS on AChR function. We demonstrate a profound reduction of whole-cell current amplitudes and prolongation of desensitization and deactivation time constants. Performing extensive functional analyses and computational modeling, we reveal decreased sodium permeability as the underlying mechanism for reduced current amplitudes in mutant AChRs. The most intriguing finding in this study, however, is the voltage-dependent conversion of ion selectivity from cationic to anionic, resulting from a single amino acid substitution. Although severely impaired, ion selectivity for sodium prevails at negative membrane potentials, whereas at positive membrane potentials, mutant AChRs become increasingly permeable for chloride.

The L251 lies at the approximate midpoint of the M2 transmembrane helix (the 9′ position, L9′) (17) and occupies a kink in each of the 5 AChR subunits pointing into the closed channel (4). Upon receptor activation, the M2 transmembrane helices rotate so that the L9′ residues no longer occlude the conduction pathway (9). In α7-neuronal AChRs, the substitution of L9′ by both polar and hydrophobic amino acids was shown to result in slower desensitization kinetics and increased sensitivity to agonist, but only substitutions with polar amino acids were associated with a change of inward rectification, which is characteristic for neuronal AChRs, into a linear I–V relationship (20). These findings are supported by studies on muscle AChRs, reporting that substitutions of L9′ by the hydrophilic amino acids serine (21) or threonine (22) in each of the 5 subunits slow desensitization and independently increase sensitivity to ACh. The substitution of L9′ by charged amino acids has not been examined so far.

In our study, the substitution of L9′ by the positively charged amino acid arginine in the α1-subunit altered AChR function in many aspects. The prolongation of desensitization and deactivation time constants is consistent with former studies (20–22). Although deactivation was not examined in those studies, its prolongation in the present study could have resulted from increased affinity of the mutant AChR for ACh as reported before (20–22). Whole-cell current amplitude was profoundly reduced, and our failure to detect single-channel currents was likely to result from single-channel amplitudes below detection level. The I–V relationship was changed into outward rectification, which was found to derive from a voltage-dependent conversion of ion selectivity from cationic to anionic. Conversion of ion selectivity was previously reported in α7-neuronal AChRs (12, 13), in 5-hydroxytryptamine_3_ receptors (14), and in glycine receptors (15), but those studies found at least 3 mutations to be necessary for successful transformation. In α7-neuronal AChRs, the conversion of ion selectivity required the insertion of proline at the cytoplasmic end of the M2 helix (between G–3′ and E–2′) along with E–2′A and V13′T (12, 13) or L9′T (12). The insertion of proline, either alone or together with E–2′A or V13′T, however, yielded nonfunctional receptors. And in the absence of the proline insertion, the E–2′ and V13′T mutations were fully compatible with cationic receptors, either when introduced alone or together (12, 13). It was hypothesized that conversion of ion selectivity in α7-neuronal AChRs is not based on alterations of the M2 helix configuration, but rather due to a local structural reorganization in the vicinity of the proline insertion (12). Data presented here provide evidence that conversion of ion selectivity does not exclusively depend on the insertion of proline between G–3′ and E–2′ but can also be induced by the substitution of L9′ by a positively charged residue. The application of USMD simulations in our study enabled us to determine the underlying mechanism of ion selectivity conversion as well as obtain an energetic description of this process. The substitution of L9′ by arginine in both α1-subunits leads to a constriction of the channel pore radius from 3.6 Å in the AChR_α1WT_ to 2.4 Å in the AChR_α1L251R_ as measured by the minimum time-averaged pore radius. This represents a 33% reduction in minimum pore diameter, requiring that ions partially desolvate in order to permeate the AChR_α1L251R_ pore (Fig. 5*D*). Ion desolvation is associated with an energetic penalty that is compensated for by the favorable electrostatic interaction of both arginines with chloride. At positive membrane potentials, when AChR_α1L251R_ conductance for chloride is high, current amplitudes are still reduced compared to AChR_α1WT_. This might be due to the strong interaction between chloride and both arginines resulting in an energetic reward for chloride with increasing dwell times at the position of both arginines translating to a reduction in conductance for chloride.

As the exact nature of the open state in Cys-loop receptors is still unclear, precisely how permeation occurs is unknown. Nevertheless, while the absolute values in the PMFs may show some variation, depending on the precise treatment of the model, the relative values are likely to be robust.

Among nicotinic acetylcholine receptors, only muscle AChRs display a linear I–V relationship (23), while neuronal AChRs are characterized by inward rectification (24). Mechanisms postulated to underlie inward rectification in neuronal AChRs include a combination of block by intracellular magnesium or polyamines and voltage-dependent mechanisms intrinsic to the receptor (25–27). In our study, outward rectification derived from an asymmetry of AChR_α1L251R_ conductance for chloride, which was high at positive membrane potentials but declined progressively as the membrane was hyperpolarized. Instead, whole-cell currents, although markedly reduced, were found to be carried by sodium at negative membrane potentials. As an underlying mechanism, the membrane potential could have influenced the relative conformation of the charged arginine residues within the channel pore, and, consequently, desolvation and the permeability of chloride.

With the AChR_α1L251R_, AChR_α1WT/α1L251R_, and AChR_α1L251R/α1WT_ representing receptors with negligible sodium conductance, only AChR_α1WT_ (accounting for around 25% of normal AChR numbers) remains to be functionally active in our patient with CMS. Thus the major pathogenic mechanism for CMS in this case is not a reduction in AChR number on the cell surface, but a reduction in the number of functional AChRs on the cell surface (with 75% of the cell surface receptors not allowing sodium ions to contribute to membrane depolarization). One would thus expect the phenotype to have similar characteristics to a mild AChR deficiency syndrome, and this is precisely what we find for this patient. A reduction of the surface expression of functional AChRs to around 25% of normal is found to be on the cusp with respect to compromising the safety margin for neuromuscular transmission (28) and is compatible with the relatively mild phenotype of the patient. A precedent for a similar effect of a heterozygous mutation in CHRNA1 has previously been demonstrated for a severe fast channel mutation (28). The conversion of ion selectivity from cationic to anionic in AChR_α1L251R_ could add a detrimental impact on endplate potentials or action potentials at the neuromuscular junction. Upon activation by ACh, the inhibitory current carried by chloride influx in AChR_α1L251R_ could antagonize the endplate potential during depolarization mediated by the remaining AChR_α1WT_. The threshold potential necessary for the generation of an action potential in muscle cells, however, ranges around −50 mV (29), which is far below the membrane potential when AChR_α1L251R_ becomes permeable for chloride. In situations of increased (compensatory) vesicle release (30–32) or high input resistance, it could be possible that the postsynaptic membrane is depolarized close to a potential region at which chloride may be able to pass through AChR_α1L251R_. Action potentials also cause depolarizations up to +30 to 40 mV (29) and may therefore induce inhibitory currents in AChR_α1L251R_, which could accelerate the decline phase and contribute to early termination of action potentials limiting their propagation.

In conclusion, the present study elucidates a mechanism for the conversion of ion selectivity from cationic to anionic, resulting from a single amino acid substitution in muscle AChRs, and is relevant for patients with CMS, as the transformation from an excitatory to an inhibitory channel might also provide a pathomechanism not linked to CMS so far.

## Methods

AChR surface expression was measured using the ^125^I-α-BuTx binding assay. Electrophysiological recordings were performed on transiently transfected HEK293 cells. USMD simulations were based on the cryogenic-electron microscopy structure of the α1 glycine receptor in an open state that was used as the template structure to generate a comparative model of the adult muscle AChR. Consent for the study on congenital myasthenic syndromes in the United Kingdom was approved by Oxfordshire Research Ethics Committee (REC) B: 04.OXB.017 and Oxfordshire REC C: 09/H0606/74, and informed consent was given by the patient in this study. Full materials and methods are available in *SI Appendix*, *Materials and Methods*.

## Supplementary Material

Supplementary File
