# Supplementary material for: Muscle acetylcholine receptor conversion into chloride conductance at positive potentials by a single mutation
Source: Proc Natl Acad Sci U S A. 2019 Sep 30;116(42):21228–35. doi: 10.1073/pnas.1908284116 (PMC6800317; doi:10.1073/pnas.1908284116)
Supplement: Supplementary File [file pnas.1908284116.sapp.pdf]

Supplementary Information for "Muscle acetylcholine receptor conversion into chloride conductance at positive potentials by a single mutation"

Hakan Cetin[a,b], Max Epstein[c], Wei W. Liu[a], Susan Maxwell[a], Pedro M. Rodriguez Cruz[a], Judith Cossins[a], Angela Vincent[a], Richard Webster[a], Philip C. Biggin[c], David Beeson[a]

[a]Nuffield Department of Clinical Neurosciences, University of Oxford, Oxford, UK

[b]Department of Neurology, Medical University of Vienna, Vienna, Austria

[c]Structural Bioinformatics and Computational Biochemistry, Department of Biochemistry, University of Oxford, Oxford, UK

Materials and Methods

Plasmids and constructs. Complementary DNAs encoding human AChR α1WT-(P3A negative isoform), β1-, δ- and ε-subunits were cloned into pcDNA3.1-hygro. The CHRNA1 mutation (c.812T>G, p. α1Leu271Arg) (transcript numbering according to ENST00000348749.9, which does not harbour the P3A exon, but contains the 20 amino acid signal sequence) was engineered into the AChR α1-subunit. For ease of comparison with the published literature this mutation is termed 'α1L251R'. α1L251R was generated using the QuikChange Site-Directed Mutagenesis Kit (Agilent Technologies) with primer 5'-GAGCATCTCTGTCTTACGGTCTTTGACTGTGTTCC-3'. The presence of the mutated residue and the absence of other variants were confirmed by Sanger sequencing. The plasmid pEGFP-N1 was used to express the fluorescent marker enhanced green fluorescent protein (EGFP). The pcDNA3.1-hygro-α1WT-GFP plasmid was generated by cloning GFP into pcDNA3.1-hygro-

α1WT using EcoRV restriction site after α1Ile348. The pcDNA3.1-hygro-α1L251R-GFP plasmid was then generated using site-directed mutagenesis kit. Both plasmids were used to express GFP-tagged α1WT- and α1L251R-subunits in western blot analyses and immunofluorescence studies.

Cell culture and transfections. HEK293 cells were maintained at 37°C and 5% CO_2 in DMEM supplemented with 10% FCS and 1% PSA. They were seeded at 10^6 cells per well in 6-well plates. At 60-80% confluence on the following day, cells were exposed to a mix of 3 µg cDNA, 20% glucose and PEI in 2 ml of growth medium. Human muscle AChR α1WT-, β1-, δ-, ε-subunit cDNA and pEGFP-N1 in a ratio of 2:1:1:1:0.2 were transfected into HEK293 cells for wild-type adult AChR expression (referred to as $AChR_{α1WT}$), α1L251R-, β1-, δ-, ε-subunit cDNA and pEGFP-N1 in a ratio of 2:1:1:1:0.2 was used for mutant AChR expression (referred to as $AChR_{α1L251R}$) and α1WT-, α1L251R-, β1-, δ-, ε-subunit cDNA and pEGFP-N1 in a ratio of 1:1:1:1:1:0.2 was used to express a mixture of AChRs with two α1WT-subunits, with two α1L251R-subunits and with one α1WT- and one α1L251R-subunit (referred to as $HEK293_{α1WT/α1L251R\_Mix}$ emphasizing the expression of AChRs with a mixed range of subunit stoichiometries; correspondingly, $HEK293_{α1WT}$ referred to HEK293 cells expressing $AChR_{α1WT}$ only, and $HEK293_{α1L251R}$ referred to HEK293 cells expressing $AChR_{α1L251R}$ only). The expression of both α1WT- and α1L251R-subunits in HEK293 cells represented the in vivo AChR expression in the heterozygous status with a mixed range of subunit stoichiometries. Theoretically, this would result in the expression of 25% $AChR_{α1WT/α1WT}$, 25% $AChR_{α1WT/α1L251R}$, $AChR_{α1L251R/α1WT}$ and 25% $AChR_{α1L251R/α1L251R}$ in individual cells. These fractions, however, can vary if the surface expression of AChRs bearing the α1L251R mutation is reduced. Complementary DNA coding for enhanced green fluorescent protein

(pEGFP-N1, Invitrogen) was included as a marker of transfection.

Cell-surface ^{125}I-α-bungarotoxin binding assay. The levels of AChRs on the surface of cells cultured in 6-well plates were measured by the ^{125}I-α-bungarotoxin (^{125}I-α-BuTx) binding assay. In HEK293 cells, the assay was performed 48 hours post-transfection. To ensure the reproducibility of results, each assay analyzed triplicate wells of cells for each condition. Each well of cells was washed three times with PBS, followed by incubations with 500 µl of ^{125}I-α-BuTx at 10^6 pm/ml diluted in blocking solution (DMEM containing 20 mM HEPES and 1% bovine serum albumin, BSA) for 1 hour at room temperature with gentle rocking. Subsequently, the cells were washed three times for 5 minutes each with 1 ml of PBS and then dissolved in 500 µl of protein extraction buffer. The cell extracts were transferred to an Eppendorf tube and the amounts of ^{125}I-α-BuTx bound were quantified by a gamma-counter. Untransfected HEK293 cells were used for the estimation of the background level of radioactivity, which was subtracted from the cell-surface ^{125}I-α-BuTx binding in transfected cells to calculate AChR expression levels.

Western blots and densitometry

HEK293 cells were seeded at 3 x 10^5 cells per well of a 6-well plate and transfected with 3 µg DNA per well the following day with a ratio of 2:1:1:1 of human muscle AChR α1WT-, β1-, δ-, ε-subunits, a 2:1:1:1 ratio of α1L251R-, β1-, δ-, ε-subunits, a 2:1:1:1 ratio of α1WT-GFP-, β1-, δ-, ε-subunits, a 2:1:1:1 ratio of α1L251R-GFP-, β1-, δ-, ε-subunits, a 1:1:1:1:1 ratio of α1WT-, α1WT-GFP, β1-, δ-, ε-subunits and a 1:1:1:1:1 ratio of α1WT-, α1L251R-GFP, β1-, δ-, ε-subunits. 48 hours post-transfection, transfected cells were lysed at 4°C for 1 hour in lysis buffer (10 mM Tris-HCl, 100 mM NaCl, 1 mM EDTA, 1% Triton-X, pH 7.5) containing 1:100 protease

inhibitor cocktail (Sigma-Aldrich, Cat# P8340). The expression of total α1- and δ-subunits were analyzed by SDS-PAGE using rabbit anti-human α1 polyclonal antibody (1:200, produced in the host laboratory) and mouse anti-human δ monoclonal antibody (1:500, Santa Cruz, Cat# sc-390896). Surface AChRs were precipitated with mouse anti-human α1 monoclonal antibody (1:200, Santa Cruz, Cat# sc-32253, RRID:AB_626626). The expression of surface α1-GFP and α1L251R-GFP were analyzed by SDS-PAGE using both rabbit anti-human α1 polyclonal antibody (1:200, produced in the host laboratory) and mouse anti-GFP monoclonal antibody (1:1000, Proteintech, Cat# 66002-1-Ig, RRID:AB_11182611), HRP-conjugated rat anti-mouse secondary antibody (1:1000, Abcam, Cat# ab131368), HRP-conjugated goat anti-rabbit secondary antibody (1:1000, Agilent, Cat# P0448, RRID:AB_2617138) and ECL (GM Healthcare). Beta-actin (1:20000, GeneTex, Cat# GTX629630, RRID:AB_2728646) was used as a loading control. Densitometry of bands was analyzed using ImageJ software.

Fluorescence microscopy

Surface AChRs were labeled with mouse anti-human α1 monoclonal antibody (1:200, Santa Cruz, Cat# sc-32253, RRID:AB_626626) 48 hours after transfection for 1 hour at room temperature, which binds the main immunogenic region of the AChR α1-subunit. Cells were washed three times with staining medium (DMEM containing 20 mM HEPES and 1% bovine serum albumin, BSA), fixed with 3% paraformaldehyde at room temperature for 10 minutes, washed three times with PBS and incubated with secondary antibody Alexa Fluor® 594 goat anti-mouse IgG (H+L) (1:750, Invitrogen, Cat# A11005, RRID:AB_141372). Cells were washed three times in PBS and mounted in fluorescence mounting medium (Dako Cytomation). Images (60x objective) were captured using Olympus IX71 fluorescence microscope with Simple

PCI (Digital Pixel).

Electrophysiology. All experiments were performed at room temperature. Single-channel recordings were performed in the cell-attached patch configuration. Recording pipettes were made of borosilicate glass (GC150F-10 Harvard Apparatus). Extracellular solution contained (in mM): NaCl 150, KCl 2.8, HEPES 10, $MgCl_2$ 2, $CaCl_2$ 2 and glucose 10 with pH adjusted to 7.4 using NaOH. Pipette solution was the same as extracellular solution, except glucose was omitted and ACh added. Single-channel currents were amplified with an Axopatch 200B amplifier (Molecular Devices), filtered at 5 kHz and sampled to hard disk at 100 kHz. The resolution was set at 50 µs. Channel transitions were detected by 50% amplitude threshold crossing (pClamp 10). For whole-cell patch clamp experiments, recording pipettes were made of thin-walled borosilicate glass (GC150TF-10, Harvard Apparatus). Pipette tips were fire-polished to a final resistance of 2-3 MΩ (MF-900 Microforge, Narishige). The extracellular solution was the same as in single-channel patch clamp experiments. The pipette solution contained (in mM): NaCl 4, KCl 144, HEPES 10, $MgCl_2$ 2, ATP 2 and EGTA 10 with pH adjusted to 7.2 using KOH. Currents were amplified using an Axopatch-1D amplifier (Molecular Devices) and, after filtering at 5 kHz, sampled to hard disk at 25 kHz. Series resistance was compensated for by at least 95%.

Fast solution exchange was accomplished using the modified HSSE-2/3 application system (ALA Scientific Instruments). A two-barrel perfusion pipette with a tip diameter of ~300 µm was used to switch between test and control solutions, which consisted of an agonist containing solution for receptor stimulation (extracellular solution with 1 mM ACh, i.e. test solution) and an agonist-free solution for agonist removal (extracellular solution, i.e. control solution). Application times were fast with 10-90% rise times < 1 ms in open pipette experiments.

Peak currents, desensitization and deactivation were measured at a holding potential of −60 mV. I-V curves were obtained by changing the holding potential from –100 mV to +100 mV by 20 mV steps. The rectification index (RI) was calculated as the peak current at positive holding potentials divided by the peak current at corresponding negative holding potentials. A RI < 1 indicates inward rectification, a RI = 1 indicates a linear I-V relationship and a RI > 1 indicates outward rectification. Data from experiments that tested different concentrations of chloride or sodium were corrected for liquid junction potentials using pClamp 10.

Homology modeling. The transmembrane domain (TMD) of adult muscle AChR α1WT- (P3A negative isoform), β1-, δ- and ε-subunits were aligned with the α1 glycine receptor TMD and other phylogenetically relevant sequences using the MUSCLE multiple sequence alignment tool (2) before being manually edited to give the final alignment (Supplementary Fig. 3). AGT linkers were then added between all M3 and M4 helices in place of intracellular domain loops. The cryo-EM structure of the α1 glycine receptor in an open state (PDB code: 3JAE) (3) was used as the template structure to generate a comparative model of the adult muscle AChR. MODELLER version 9.12 (4) was used to initially generate 100 models that were assessed and selected via examination of their GA341 and DOPE scores. The α1L251R mutations were made in PyMol (The PyMOL Molecular Graphics System, Version 1.4, Schrödinger LLC). As the exact nature of the open state in Cys-loop receptors is still unclear, precisely how permeation occurs is unknown. Nevertheless, whilst the absolute values obtained in the PMFs may show some variation depending on the precise treatment of the model, the relative values are likely to be robust, as supported by the wild-type values obtained in the control PMF profiles.

Simulation set up. All molecular dynamics simulations were performed with GROMACS (5). Models, represented by the AMBER ff99sb-ildn (6) force-field, were embedded into a pure POPC bilayer using the inflategro.pl protocol (7) and then solvated with TIP3P water model (8). Charges were neutralized before subsequent addition of 0.15 M NaCl. The resulting system was then energy minimized according to the steepest descent algorithm. Particle mesh Ewald algorithm (9) was used for computing electrostatics. The LINCS algorithm (10) was used to constrain H-bonds thereby allowing for a time step of 2 fs.

Equilibration was performed initially for 1 ns with V-rescale temperature coupling (11) with tau set to 0.1 ps and a Berendsen barostat (12) with a tau value of 1 ps for pressure coupling of the system. Further equilibration was conducted for 4 ns using the Nose-Hoover (13) and Parrinello-Rahman (14) temperature and pressure coupling algorithms with respective tau values of 0.5 and 1 ps. Protein backbone atoms were restrained during equilibration with a force constant of 1000 kJ/mol/nm^2. Production runs were conducted in the NPT ensemble using V-rescale temperature coupling τ_T 0.1 and Parrinello-Rahman pressure coupling τ_P 1.0. Simulations were conducted at 310 K.

Potential of mean force (PMF) calculations were performed whereby the reaction coordinate (*z*) was defined as the distance along the pore with respect to the center of protein mass (*z* = 0) with values ranging to/from ±40 Å. Windows were positioned with 0.5 Å and 1 Å spacing, with the closer windows positioned at sharp energy barriers. Starting coordinates for each window were minimized as described above.

Additional PMFs for probe ions in bulk solution were obtained in order to determine the relaxation time of the system (Supplementary Fig. 4). Energy value tolerance was within thermal energy after approximately 3 ns and simulation data

before then was therefore discarded. To ensure that the model remained in the open state (as defined by the GlyR template), protein backbone atoms were restrained with a harmonic force-constant of 1000 kJ/mol/nm^2. Additional windows were run at saddle points in the PMF, with a force constant of 500 kJ/mol/nm^2 to ensure good histogram overlap between regions. The weighted histogram analysis method was used in order to calculate the potential of mean force (15) with five bootstraps used for estimation of statistical uncertainty. Solvation analysis was conducted using the MDAnalysis (16) python package and molecular visualizations made using Visual Molecular Dynamics (VMD) (17) and PyMol version 1.4 (The PyMOL Molecular Graphics System, Version 2.0, Schrödinger LLC).

Block analysis of central barrier height convergence. After discarding the first 3 ns of simulation time as equilibration, a block analysis was performed with incrementally large data sets to determine if convergence of PMFs had occurred. All profiles converged successfully after 2 ns.

Statistical analysis. All data were analyzed using GraphPad Prism v7.0a. Results are presented as means ± standard error of the mean, with n referring to the number of cells. Statistical analyses were performed using an unpaired t-test for the comparison of two groups ($p < 0.05$, two-sided), and using a one-way ANOVA for the comparison of more than two groups ($p < 0.05$, two-sided). Significant main effects were followed by Tukey's multiple comparison test. Clampfit 10.5 was used for exponential curve fitting and current decay time constant calculation.

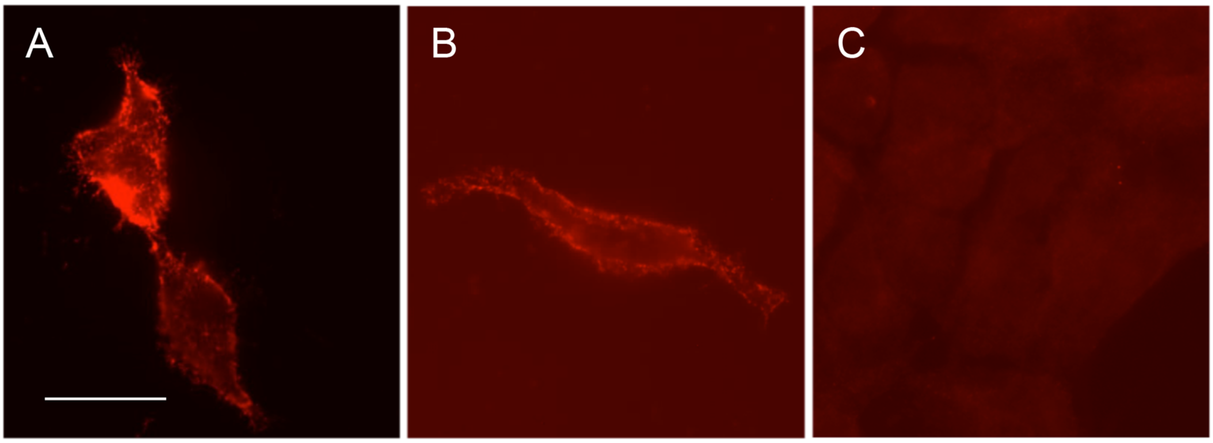

Supplementary Figure 1

Binding of the mouse anti-human α1 monoclonal antibody to surface AChR$_{\alpha 1WT}$ and AChR$_{\alpha 1L251R}$

HEK293$_{\alpha 1WT}$ (A), HEK293$_{\alpha 1L251R}$ (B) and untransfected HEK293 cells (C) were labeled with a mouse anti-human α1 monoclonal antibody and with a secondary goat anti-mouse Alexa Fluor® 594 nm antibody. Scale bar = 20 μm.

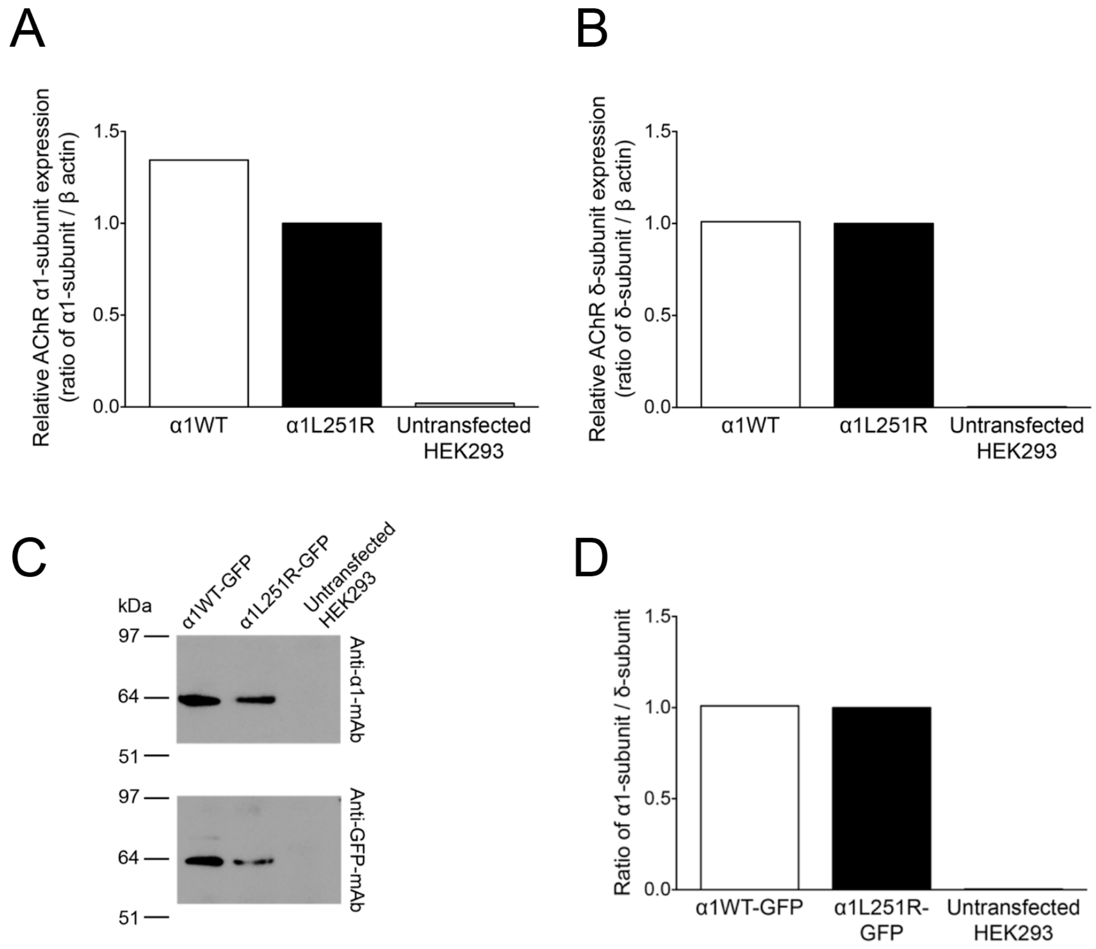

Supplementary Figure 2

Densitometry analyses of AChR whole-cell extracts and surface AChR$_{\alpha 1WT}$ and AChR$_{\alpha 1L251R}$ expression

Densitometry analyses of whole-cell extracts corresponding to the western blots in Fig. 1*B* in the manuscript with quantification of the relative α1WT- or α1L251R-subunit expression (A) and the relative δ-subunit expression (B). The bars indicate the ratio between the corresponding subunit and β actin. (C) Western blots of immunoprecipitated surface AChRs using a mouse anti-human α1 monoclonal antibody (top) and a mouse anti-GFP monoclonal antibody (bottom), respectively. HEK293 cells were transfected with a 2:1:1:1 ratio of α1WT-GFP-, β1-, δ-, ε-subunits (left lane) or with a 2:1:1:1 ratio of α1L251R-GFP-, β1-, δ-, ε-subunits (middle lane). (D) Densitometry analysis corresponding to the western blot in Fig. 1*C* in the

manuscript with quantification of the α1WT-/δ-subunit or the α1L251R-/δ-subunit ratio.

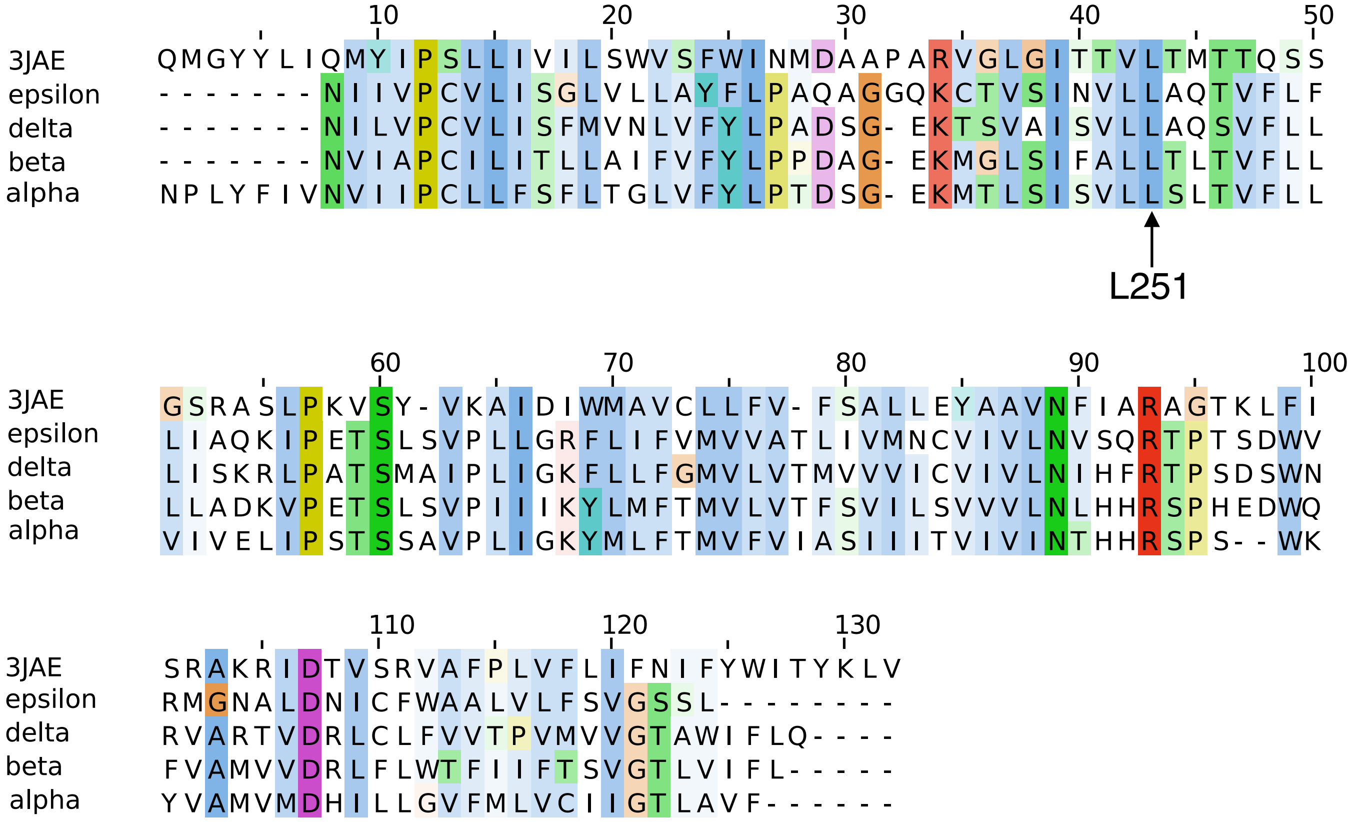

Supplementary Figure 3

Multiple sequence alignment for the adult muscle AChR

Sequences are displayed in Jalview with the ClustalX coloring scheme to show conserved regions.

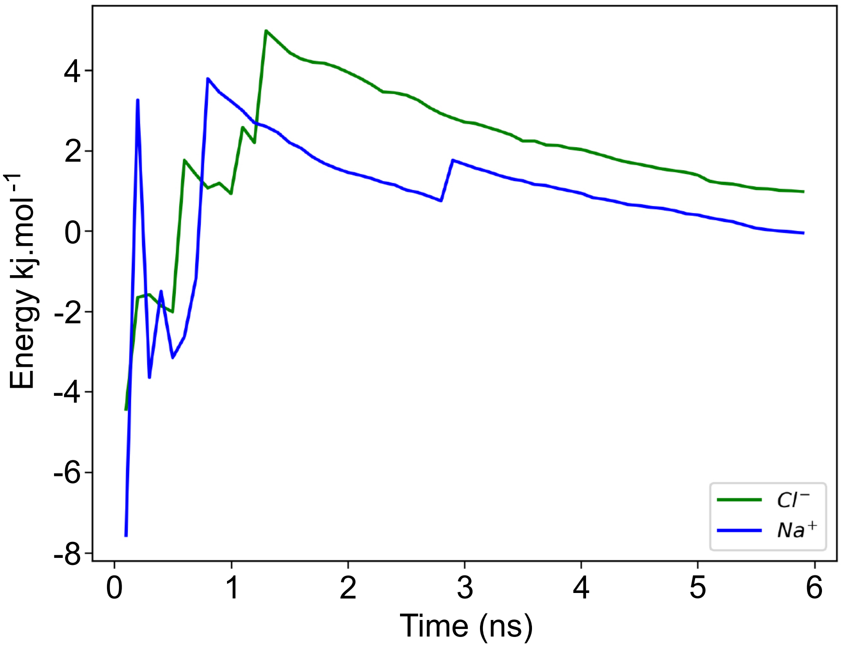

Supplementary Figure 4

Energy convergence of ions in bulk water

PMFs were obtained for probe ions in bulk water, an average value was then taken across the PMF using 0.1 ns blocks to assess values.

References

1. Jacobson L, Beeson D, Tzartos S, & Vincent A (1999) Monoclonal antibodies raised against human acetylcholine receptor bind to all five subunits of the fetal isoform. *J Neuroimmunol* 98(2):112-120.
2. Edgar RC (2004) MUSCLE: a multiple sequence alignment method with reduced time and space complexity. *BMC Bioinformatics* 5:113.
3. Du J, Lu W, Wu S, Cheng Y, & Gouaux E (2015) Glycine receptor mechanism elucidated by electron cryo-microscopy. *Nature* 526(7572):224-229.
4. Eswar N*, et al.* (2006) Comparative protein structure modeling using Modeller. *Curr Protoc Bioinformatics* Chapter 5:Unit-5 6.
5. Abraham MJ*, et al.* (2015) GROMACS: High performance molecular simulations through multi-level parallelism from laptops to supercomputers. *SoftwareX* 1-2:19-25.
6. Lindorff-Larsen K*, et al.* (2010) Improved side-chain torsion potentials for the Amber ff99SB protein force field. *Proteins* 78(8):1950-1958.
7. Kandt C, Ash WL, & Tieleman DP (2007) Setting up and running molecular dynamics simulations of membrane proteins. *Methods* 41(4):475-488.
8. Jorgensen WL, Chandresekhar J, Madura JD, Impey RW, & Klein ML (1983) Comparison of simple potential functions for simulating liquid water. *J Chem Phys* 79(79):926-935.
9. Darden T, York D, & Pedersen L (1993) Particle mesh Ewald: An N.log(N) method for Ewald sums in large systems. *J Chem Phys* 98:10089-10092.
10. Hess B, Bekker H, Berendsen HJ, & Fraaije JGEM (1997) LINCS: A linear constraint solver for molecular simulations. *J Comput Chem* 18:1463-1472.
11. Bussi G, Donadio D, & Parrinello M (2007) Canonical sampling through velocity rescaling. *J Chem Phys* 126(1):014101.

12. Berendsen HJC, Postma JPM, van Gunsteren WF, DiNola A, & Haak JR (1984) Molecular dynamics with coupling to an external bath. *J Chem Phys* 81:3684-3690.
13. Nosé S (1984) A molecular dynamics method for simulations in the canonical ensemble. *Mol Phys* 52:255-268.
14. Parrinello M, Rahman A (1981) Polymorphic transitions in single crystals: A new molecular dynamics method. *J Appl Phys* 52:7182-7190.
15. Kumar S, Rosenberg JM, Bouzida D, Swendsen RH, & Kollman PA (1992) The weighted histogram analysis method for free energy calculations on biomolecules. I: The method. *J Comput Chem* 13:1011-1021.
16. Michaud-Agrawal N, Denning EJ, Woolf TB, & Beckstein O (2011) MDAnalysis: a toolkit for the analysis of molecular dynamics simulations. *J Comput Chem* 32(10):2319-2327.
17. Humphrey W, Dalke A, & Schulten K (1996) VMD: visual molecular dynamics. *J Mol Graph* 14(1):33-38, 27-38.
